# A Dual Route Model of Empathy: A Neurobiological Prospective

**DOI:** 10.3389/fpsyg.2018.02212

**Published:** 2018-11-13

**Authors:** Chi-Lin Yu, Tai-Li Chou

**Affiliations:** ^1^Department of Psychology, National Taiwan University, Taipei, Taiwan; ^2^Center for Advanced Study in the Behavioral Sciences, Stanford University, Stanford, CA, United States

**Keywords:** empathy, affective empathy, cognitive empathy, dual routes, social neuroscience

## Introduction

Human beings are physically independent but are psychologically connected. We spend a lot of time dealing with others' intentions, experiences, and internal states. The term empathy describes these phenomena. Previous studies defined empathy as “the ability and tendency to share and understand others' internal state” (Zaki and Ochsner, 2012). Consistent with this definition, accumulated empirical evidence showed that empathy is a multifaceted construct composed of two components, including affective empathy and cognitive empathy (Hoffman, 1984; Decety and Jackson, 2004; Singer, 2006; Uddin et al., 2007; Shamay-Tsoory et al., 2009; Barrett et al., 2016).

Recently, ever-growing studies have considered empathy under different viewpoints (e.g., developmental trajectories, naturalism, disordered population, and so on), and have summarized the independence and non-independence of affective empathy and cognitive empathy (Barrett et al., 2016). These studies provide significant contributions to the progress of empathy research. However, we are not yet to fully understand the neurocognitive mechanisms between the affective empathy and cognitive empathy. Three major limitations restrict our knowledge toward empathy. First, it is rare to characterize cognitive and affective empathy in terms of processing speed and involvement of consciousness. Second, previous research often studies cognitive and affective empathy separately. It is hard to identify and explain the interactive nature between these two components. Third, influential factors, including attention and prior knowledge, have not yet been considered in the framework of empathy, thus under-estimating their impacts on empathy processing. Attention and prior knowledge are closely linked with the two aforementioned limitations, specifically processing speed and consciousness involvement as well as the integration of affective empathy and cognitive empathy. Thus, these two factors are discussed in the present opinion. To address these limitations, the present opinion proposes a novel and general framework to summarize both behavioral and neural evidence in the literature. The proposed dual route model of empathy is mainly composed of an *automatic, fast*, and *specific* “lower route” with affective empathy as well as a *complex, slow*, and *iterative* “higher route” with cognitive empathy. This proposed empathy model aims to integrate these two routes and to include the influences of attention and prior knowledge.

Previously, in the domain of emotion neuroscience, Joseph LeDuox, a pioneer to study emotions, proposed a framework with two parallel neural systems, namely a low road and a high road (LeDoux, 1998). Specifically, the low road, which is a fast, subcortical, short-latency pathway with minimal cortical involvement, directly conveys sensory information from the thalamus to the amygdala, allowing stimuli to be processed automatically without consciousness and awareness (Davis, 1992; LeDoux, 1995, 1997). These features of rapid and automatic processes construct the low road emotion processes. In contrast, the high road indicates the visual pathways from retina neuron to visual cortex, and then connects to inferior temporal lobe for processing higher level consciousness of emotion feeling, and finally directs to the amygdala (LeDoux, 1998). These features of slow processing and more involvement of consciousness establish the high road of emotion.

**Figure 1:**
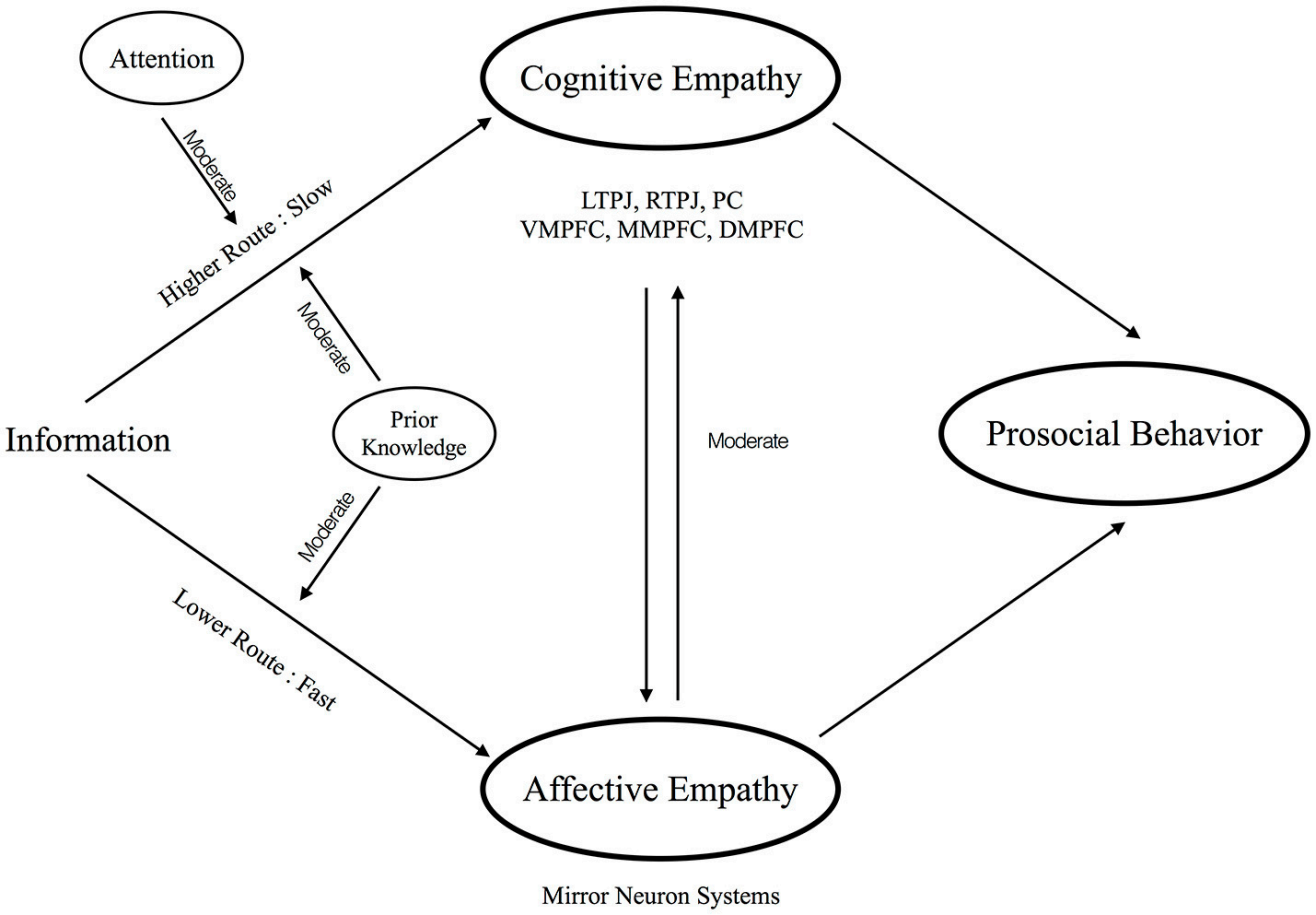
The conceptual visualization for the proposed dual route model of empathy.

The present opinion hypothesizes that empathy also has a similar dual route system, which includes an automatic, fast and lower-level route (i.e., lower route) and a complex, slow and higher-level route (i.e., higher route), inside our brain (Figure 1). The rest of the paper is organized as a series of introductions for each component of empathy. Also, we consider these components in the proposed dual route model of empathy in order to obtain the whole picture of the empathy processing.

## Lower route: affective empathy

First of all, the lower route is an efficient, automatic and fast process with minimal involvement of consciousness. Affective empathy, which defined as one's emotional, sensorimotor, and visceral response to the affective state of other, encompasses the mentioned-above efficient, automatic and fast features of the lower route. Affective empathy is also usually described by different but aligned terms like “experience sharing” (Zaki and Ochsner, 2012; Barrett et al., 2016) or “empathic concern” (Davis, 1994).

From the perspective of behavioral studies, affective empathy captures the phenomena that people automatically share the experiences with the targets they observe, such as arousal (Vaughan and Lanzetta, 1980; Levenson and Ruef, 1992), moods (Neumann and Strack, 2000) and facial expression (Dimberg and Thunberg, 1998). It has long been shown that affective empathy can rapidly occur (Dimberg and Thunberg, 1998), even outside of our consciousness and awareness (Neumann and Strack, 2000). It is also suggested that affective empathy quickly emerges and appears stable in early development (Knafo et al., 2008; Roth-Hanania et al., 2011; Davidov et al., 2013). For example, infants express emotional feeling while hearing the sound of other's cries rather than hearing their own cries (Sagi and Hoffman, 1976; Martin and Clark, 1982) and are able to respond to other's facial expressions in the first few weeks of life (Haviland and Lelwica, 1987). Overall, behavioral findings support the idea that affective empathy is a basic and primitive beginning of empathy (Hoffman, 2001).

From the viewpoints of neural evidence, research studying affective empathy over two decades consistently reveal a specific system, the mirror neuron system, as the underpinning of experience sharing processes (Di Pellegrino et al., 1992; Iacoboni, 2009; Rizzolatti and Sinigaglia, 2010, 2016). The mirror neuron system is thought to comprise the inferior frontal cortex, the premotor areas, and the insula. When we observe that others are experiencing some sensorimotor or affective information, our mirror neuron system provides a simple mechanism to generate the representations of other's states by simulating neuronal activities similar to the observed ones, thus allowing an automatic share of others' experiences (Iacoboni et al., 2005; Heyes, 2011). Furthermore, regions in the mirror neuron system demonstrate automatic (Iacoboni et al., 2005; Heyes, 2011) and unconscious information transition (Carr et al., 2003; Leslie et al., 2004). Moreover, the neural profiles of affective empathy are specifically located in the mirror neuron system with little requirements of additional cortical regions.

Supported by both behavioral and neural evidence, affective empathy, which includes basic, unconscious, automatic and fast processes, constructs the lower route in the model.

## Higher route: cognitive empathy

The second route in the model is the higher route, which is a slow and complex process with efforts, consciousness, and elaborated neural profiles. Cognitive empathy, which refers to the ability to understand or explicitly reason the subjective mental states, perspectives or intentions of others (Gopnik and Wellman, 1992), establishes the higher route of the model. Cognitive empathy is also known as “mentalizing” (Barrett et al., 2016), “Theory of Mind” (Premack and Woodruff, 1978) or “perspective taking” (Davis, 1994).

From the perspective of behavioral findings, several demonstrations suggest that cognitive empathy is effortful (Lin et al., 2010) and requires attention and time (Gilovich et al., 2000; Keysar et al., 2000). People would make wrong inferences on the internal states of others because of the attentional disruption or limited time. In addition, developmental findings indicate that cognitive empathy, which relies on the involvement of other cognitive abilities (e.g., inhibition, execute functions), does not appear until the first year (Onishi and Baillargeon, 2005). Also, an anchoring and adjustment mechanism of cognitive empathy is proposed to describe its complex processing (Gilovich et al., 2000; Barrett et al., 2016). Namely, people first use their awareness and effort to establish personal theories or assumptions about others' psychological states, and then further adjust and correct the generated theories during interactions. In brief, all the arguments provided by the behavioral literature suggest a more elaborated, complex, and high-level processing of cognitive empathy (Carlson and Moses, 2001; Wellman et al., 2001).

From the viewpoints of neuroscience research, imaging studies also reveal a complete neural profile for cognitive empathy. Engaged brain regions mainly include dorsal, middle, and ventral medial prefrontal cortex (DMPFC, MMPFC, VMPFC), precuneus (PC), and temporoparietal junction (TPJ). These areas are selectively activated when subjects make inferences on the information about others' mental states. The impairments of cognitive empathy are also highly correlated with atypical activity of these regions. For example, individuals with autism spectrum disorders (ASD), who have difficulties in cognitive empathy, often exhibit altered brain patterns in cognitive empathy-associated regions in rest (Picci et al., 2016; Hull et al., 2017) or under cognitive processing (Philip et al., 2012; Maximo et al., 2014; Picci et al., 2016). Rather than depending on a single system as shown in the lower route, cognitive empathy requires the involvements of a variety of brain regions (i.e., TPJ, PC, VMPFC, MMPFC, and DMPFC). These regions also demonstrate a complex hierarchical structure (Van Overwalle and Vandekerckhove, 2013). That is, the TPJ processes the inferences of others' intentions and belief, the MPFC associates with the inferences of others' traits or stable characteristics, and the iterative re-processing between TPJ and MPFC further extracts the information of others' mental states (Van Overwalle and Baetens, 2009; Van Overwalle and Vandekerckhove, 2013). This slow, complex, and iterative processing echoes the aforementioned anchoring and adjustment mechanism.

Moreover, we hypothesize attention to moderate the higher route. As mentioned, participants could not correctly infer others' belief or knowledge when distracted (Gilovich et al., 2000). Further studies even argued that people appear to become “mindblind,” meaning that they fail to mentalize others when they have no sufficient attention resource (Lin et al., 2010). Together, these findings not only state the effortful and complex process in the higher route, but also highlight the moderating role of attention on the higher route.

Evidenced by arguments from behavioral and neural findings, cognitive empathy with slow, complex and higher-level features is the essence of the higher route in the empathy framework.

## Hypothesized connections

In addition to the aforementioned two routes constructing the main structure of the dual route model of empathy, several hypothesized connections are proposed to elaborate the relationships between two routes.

First, the connections between affective and cognitive empathy may have influences on each other, thereby impacting the level of empathy processing. On one hand, there may be a modulation from the lower route to higher route. For example, our ability to mentalize others depends on whether we have shared their feelings. On the other hand, a modulation from the higher route to lower route may exist. For instance, when we fully know about others' mind, we probably can be more able to know others' feeling. Neural evidence echoes this speculation by suggesting the influences of connectivity between regions of affective and cognitive empathy on social abilities (Fishman et al., 2014; Libero et al., 2014). Also, a behavioral study with ASD supported this argument by suggesting that affective empathy and cognitive empathy are inter-dependent (Bos and Stokes, 2018). Whilst it is a topic under investigations, the present opinion highlights an interactive connection between two routes.

Second, our prior knowledge, such as impression and familiarity about others, may affect the processes in the lower and higher route (Han and Northoff, 2008; Serino et al., 2009; Xu et al., 2009; Liew et al., 2011). For example, previous evidence showed that both cognitive and affective empathy-associated brain regions are more activated when people observe familiar friends than unfamiliar strangers in pain (Meyer et al., 2012). A number of studies further suggested that the brain regions for impression formation are highly overlapped with the areas supporting cognitive empathy (Mitchell et al., 2004; Schiller et al., 2009; Yu et al., 2016). According to these lines of evidence, the present opinion suggests that our prior knowledge constructs a moderated role on both the lower and higher routes.

Third, prosocial behavior is the output that flows from the two routes. That is, people who share and understand others' mind will finally care about others and generate desires to help others (Barrett et al., 2016). Supporting evidence connects both affective and cognitive empathy with prosocial behavior (Snyder and Lopez, 2009). For example, brain regions of affective empathy (Singer et al., 2008; Hein et al., 2010; Masten et al., 2011) and cognitive empathy (Rameson et al., 2012; Waytz et al., 2012) can both predict willingness to perform prosocial behaviors. Furthermore, affective empathy and cognitive empathy may have different effects on prosocial behavior. On the one hand, since affective empathy is a fast and automatic route without involvements of consciousness, it is most often elicited by explicit cues such as facial expression. In consequence, the processes of affective empathy toward prosocial behavioral can be skewed by the accessibility of the target. When a person can directly assess the cues from others, he/she can elicit more affective empathy and produce more prosocial behavioral. On the other hand, because the involvement of consciousness, cognitive empathy can support prosocial behavior but not always do so. For example, people may not show prosocial behavior to their enemies, although people can still mentalize them. In brief, integration of these two routes can affect prosocial behaviors, and is by no means monotonic.

In short, although few studies shed light on these connections, the present opinion considers them in the model as influential factors underlying the complex processes of empathy.

## Conclusions

The relationship between the higher route and lower route is closely connected. By integrating both behavioral and neural evidence, the present opinion proposes a general framework, the dual route model, as a mechanism to explain the underlying process of empathy.

## Author contributions

All authors listed have made a substantial, direct and intellectual contribution to the work, and approved it for publication.

### Conflict of interest statement

The authors declare that the research was conducted in the absence of any commercial or financial relationships that could be construed as a potential conflict of interest. The reviewer, QL, and handling Editor declared their shared affiliation.
